# Endometrial Cancer Related to Endometrial Ablation: A Narrative Review

**DOI:** 10.3390/cancers18081290

**Published:** 2026-04-19

**Authors:** George A. Vilos, Angelos G. Vilos, Meryl Hodge, Ayman Oraif, Faisal Khalid Idris, Jacob McGee, Artin Ternamian

**Affiliations:** 1 Department of Obstetrics and Gynaecology, Schulich School of Medicine & Dentistry, Western University, London, ON N6A 5C1, Canada; agvilos19@gmail.com (A.G.V.); meryl.hodge@lhsc.on.ca (M.H.); drfaisalidrees@hotmail.com (F.K.I.); jacob.mcgee@lhsc.on.ca (J.M.); 2 London Health Sciences Centre, 800 Commissioners Rd East., London, ON N6A 5W9, Canada; 3 Consultant Obstetrics & Gynecology & IVF, King’s College Hospital London, Jeddah 23423, Saudi Arabia; ayman.oraif@kch.sa; 4 Department of Obstetrics and Gynaecology, Saint Joseph’s Health, University of Toronto, Toronto, ON M5G 1E2, Canada; artin.ternamian@utoronto.ca

**Keywords:** endometrial ablation, endometrial cancer, endometrial neoplasia, uterine neoplasia, diagnosis, endometrial biopsy, hysteroscopy, delayed diagnosis

## Abstract

Persistent post-endometrial ablation uterine bleeding indicates that no method of EA eliminates the entire endometrium, and post-EA hysteroscopy shows a distorted and scarred uterine cavity in the majority of patients. These observations raise concerns regarding presentation, assessment and stage of potential post-ablation endometrial cancer (PAEC), developing in residual endometrium pockets. To better understand these concerns, a literature search was conducted, from the introduction of EA in the 1980s through 2025, to capture reports of endometrial cancer (EC) associated with or following EA using multiple data bases, imputing search terms of EC following EA and possible combinations of first- and second-generation EA techniques associated with EC. Upon review of all publications, we identified 86 ECs associated with EA, described in 20 case reports (N = 20), four case series (N = 18), eleven cohort studies (N = 21), one registry (N = 27) and five reviews. Based on 12 relevant studies at a median follow-up of 8.5 years (range 1.9–25), 43 EC were identified in 39,795 women with a history of EA, with a summary incidence of 0.11% (range 0.0–1.59%). Although the studies and data are very heterogeneous, it appears that EA may afford a protective effect in reducing the risk of EC in the short term. The mechanistic effect is likely due to a quantitative reduction in the endometrium that can potentially become malignant, and/or due to the elimination of occult pre- or malignant endometrial elements which are vulnerable to EA. Moreover, based on 25 evaluable cases, the mode and time to presentation, the diagnostic work-up (including endometrial biopsy and hysteroscopy), and the stage of PAEC appear not to be altered by EA.

## Simple Summary

Persistent uterine bleeding following endometrial ablation (EA) indicates that all contemporary EA methods fail to eliminate the entire endometrium, and hysteroscopy shows a distorted and scarred uterine cavity in the majority of women. These observations raise concerns regarding presentation, assessment and stage of potential post-ablation endometrial cancer (PAEC), developing in residual endometrial islands. To address these concerns, we conducted a literature search using multiple data bases from the 1980s, when EA was introduced, to 2025 to capture reports of endometrial cancer (EC) associated with or following EA. Based on 12 cohort studies, at a median follow-up of 8.5 years (range 1.9–25), 43 ECs were identified in 39,795 women with a history of EA, with a summary incidence of 0.11% (range 0.0–1.59%). Based on 25 evaluable cases, the mode and time to presentation, investigation, diagnosis, and stage of PAEC were not altered by EA. These publications suggest that EA may reduce EC risk in the short term, possibly due to a quantitative reduction in endometrial volume and elimination of occult pre- or malignant endometrial foci, which are vulnerable to ablation techniques.

## 1. Introduction

Endometrial ablation (EA) refers to the destruction or elimination of the endometrium using thermal (heat or cold) energy or electro-mechanical removal. It was introduced in the 1980s as a less invasive, safer and less skill-dependent alternative to hysterectomy, for women with abnormal uterine bleeding (AUB) of benign pathology, who were unwilling to attempt, did not tolerate or failed traditional medical-conservative therapy. Despite the application of different methods of EA, post-EA uterine bleeding patterns indicate that, in the majority of women, no currently available method of EA eliminates the entire endometrium. Consequently, concern has been raised regarding the incidence, timely assessment and diagnosis of potential post-ablation endometrial cancer (PAEC), due to incomplete elimination of the endometrium, leaving endometrial nest(s) which could result in sequestered island(s) of PAEC [1].

Furthermore, post-EA hysteroscopy indicates that EA causes significant distortion and scarring of the uterine cavity, impeding and/or hindering detection of sequestered island(s) of PAEC, and possibly delaying diagnosis by preventing uterine bleeding, which is a common clinical hallmark of EC, that prompts investigation [2]. Therefore, PAEC in a scarred and distorted uterine cavity may not declare itself with uterine bleeding and remain inaccessible to standard investigations, be it by endometrial sampling, biopsy and/or hysteroscopy, thus concealing and/or delaying diagnosis, upstaging the disease and probably worsening the prognosis.

Conversely, it has been argued that EA might reduce the risk of EC, since it reduces quantitatively endometrial volume that can potentially become malignant or by eliminating existing occult pre- or malignant elements, which are vulnerable to EA [3,4,5].

PAEC concerns are underscored by the fact that EC is the most common gynecologic malignancy and it is strongly associated with obesity, which has presently reached epidemic proportions [6]. Notably, the estimated relative risk for EC in women with obesity follows a positive correlation reaching an odds ratio of approximately 20 in women with BMI ≥ 40 [7]. Additionally, Mendelian randomization analysis, including all reported risk factors of EC, indicates that for every five extra BMI units, EC increases by 88%, a much larger increased risk than that reported in conventional observational studies and meta-analyses [8].

Therefore, the changing global landscape of increased prevalence of obesity in women and its strong association with increased risk of EC [7,8], together with the widespread utilization of EA to treat AUB, raises looming challenges as healthcare providers may encounter large cohorts of obese and non-obese women who had EA entering their sixth and seventh decades of life—peak years for EC.

In this narrative review, we examined publications describing EC associated with or following EA to determine incidence; patient characteristics; mode and time to presentation; diagnostic work-up, including endometrial biopsy and hysteroscopy; and stage of PAEC at diagnosis and hysterectomy.

## 2. Methods

We conducted a literature search for English language reports of EC associated with EA using MEDLINE, Google Scholar, PubMed, EMBASE and Cochrane Library data bases from the inception of EA in the 1980s through 2025. We used keywords and search terms of EC after EA, EC associated with EA, post-EA EC, post-EA endometrial biopsy, and post-EA hysteroscopy. Relevant publications included 20 case reports (N = 20) [9,10,11,12,13,14,15,16,17,18,19,20,21,22,23,24,25,26,27], four case series (N = 18) [28,29,30,31], twelve cohort studies (N = 21) [4,32,33,34,35,36,37,38,39,40,41,42], one National registry (N = 27) [43] and five reviews [2,25,44,45,46] providing information on 86 EC associated with EA.

## 3. Results

### 3.1. Incidence of PAEC

An estimated summary incidence of PAEC, constructed from 11 cohort studies [4,32,33,34,36,37,38,39,40,41,42] and one national registry [43], is 0.11% with a wide range from 0.0 to 1.59% at a median follow-up of 8.5 years (range 1.9–25) (Table 1). We excluded one additional cohort study reporting incidence 0.11% (4 EC/3769 patients) because three patients had trans-cervical resection of the endometrium (TCRE) for postmenopausal bleeding (PMB) while taking hormone replacement therapy (HRT) and had preoperative risk factors for EC including obesity, hypertension and endometrial polyps during TCRE. The fourth patient had selective resection of polyps and curettage only without having EA [35].

### 3.2. Circumstances, Patient Characteristics, Diagnosis and Stage of EC Associated with EA

Of the 86 EC cases, 43 cases provided sufficient patient information to determine the circumstances, patient characteristics, diagnosis and stage of EC associated with EA and are divided into three groups: (1) presence or likely presence of EC during EA (Table 2, N = 18); (2) EA performed in women with PMB (Table 3, N = 9); and (3) EA performed in pre-menopausal women with AUB (Table 4, N = 16).

Presence EC during EA: Table 2 includes 12 women who already had EC present during EA [9,17,28,30]. Of these, seven had benign, two proliferative and one each secretory, simple hyperplasia (non-atypical endometrial hyperplasia, NAEH) and unknown pre-EA endometrial pathology. The time from pre-EA biopsy to EA was within 3 months in six, unknown in four, and 11 and 17 months in two cases. In two cases, hysterectomy and bilateral salpingo-oophorectomy (HBSO) were performed at 34 and 60 months after EC diagnosis and the corresponding stages were IA, G1 and IA, G2, respectively. No residual EC was found in nine (75%) cases.

Likely presence of EC during EA: In three additional cases, EC was likely present during EA [12,15,20]. In one woman having EA for PMB, pre-EA biopsy was adenomatous endometrial hyperplasia (NAEH) [15] while the other two had complex endometrial hyperplasia (NAEH) [12,20]; the latter upgraded to atypical endometrial hyperplasia (AEH) during EA one month later [20]. All three cases had persistent post-EA uterine bleeding and stage 1A, G1 EC was diagnosed with endometrial biopsy and hysterectomy within 6 months of EA.

Presence of atypical endometrial hyperplasia (AEH) during EA: There were three additional cases of AEH identified during EA, with pre-EA biopsy showing insufficient, adenomatous and complex hyperplasia [11,14,16]. In two women, EA was performed for PMB. The BMI was >40 in two and unknown in one. The patient with complex hyperplasia was given Danazol (Cyclomen) 800 mg daily for 4 weeks and the TCRE specimen was reported as benign. However, the original pre-EA biopsy was re-examined and upgraded to AEH following hysterectomy for heavy PMB 3 years post-EA showing EC (stage IB, G2) [16]. AEH is a pre-malignant lesion or may coexist with EC in a significant number of women, and it should be treated like EC. However, in one patient no treatment was offered after the TCRE showed AEH. At 1-year post-EA, she had spotting, and Pap smear identified EC cells and EC (stage IA, G1) was confirmed following hysterectomy [11]. A 63-year-old with BMI > 40 refused hysterectomy after AEH, presented with umbilical metastasis of EC (stage IV) 14 months later, and died 4 months post hysterectomy [14].

PAEC in women having EA for PMB: Nine PAEC were diagnosed in women who had TCRE (N = 7), one REA and one dilation and curettage (D&C) only for PMB (Table 3) [13,21,29,31]. Eight women had risk factors for EC, with two taking combined HT and one taking progesterone only. Pre-EA biopsy was benign in seven and non-atypical endometrial hyperplasia (NAEH) in two women. During EA, pathology was benign in four, unknown in three and NAEH in two women. At a median follow-up of 6 years (range 3–10), seven (77.8%) women presented with vaginal bleeding, one with pain without bleeding and one with amenorrhea and urinary stress incontinence (USI). Hysteroscopy and D&C/biopsy diagnosed EC in six (75%). It was unsuccessful in one and not attempted in the other two women. Two PAEC were found after hysterectomy: one for USI and one for pain without bleeding. Seven PAEC (77.8%) were stage IA-IC and two were stage III (22.2%).

### 3.3. PAEC in Pre-Menopausal Women with Appropriate Indications for EA

Excluding the 27 cases (Table 2 and Table 3) and the 43 cases because of insufficient patient information, there remained 16 cases with pertinent information on patient characteristics, circumstances, and appropriate indications for EA in pre-menopausal women with AUB (Table 4) [10,18,22,23,24,25,26,27,29,31,42].

Patient characteristics and demographics: The median age and (range) at the time of EA was 47.5 years (range 33–51). The BMI was not reported in five and it was <30 in five, ≥30–<39 in five, ≥40 in one woman. Comorbidities were not provided in six women, while at least one of hypertension, diabetes, colon and breast cancer was reported in seven. No risks were reported in three women. Pre-EA endometrial biopsy was reported as benign in three, proliferative in five (one taken 1 year prior to EA), secretory in three, NAEH in two (one adenomatous, one simple) and not provided in three women. The method of EA was reported as coagulation or TCRE in eight, bipolar radiofrequency in four and TBEA in four women. Endometrial pathology during EA was not reported in 11 cases. It was proliferative in four and benign in one woman.

Presentation of PAEC: The presentation of PAEC was vaginal bleeding in 15 (93.8%); five patients had pain associated with bleeding and 10 women were postmenopausal. The woman with no post-EA bleeding received a hysterectomy for intense pain 30 months post-EA and EC (stage IB, G1) was found. The median time to presentation was 6.5 years, with a range of 1 to 18.

Diagnosis of PAEC: Diagnosis of PAEC was achieved in 14/15 (93.3%) attempted procedures using endometrial biopsy in five, D&C in one, hysteroscopy and biopsy in four and TCRE in four women. PAEC was diagnosed after hysterectomy in two women, one after a failed biopsy and the other had a hysterectomy for intense pain without bleeding.

Stage of PAEC: PAEC was reported as stage I in 13 (81%), stage II/III in two (1.3%), and unknown stage in one woman.

## 4. Discussion

*Incidence of PAEC:* In our narrative review, three studies using background controls found no statistically significant difference in risk reduction in EC after EA, likely due to inadequate power [33,39,41]. Statistically significant incidence reduction was reported by two studies [incidence 0.08%, (0.0–0.22)] [4,42]. A large registry found a significant reduction after REA [standardized incidence ratio SIR 0.53 (0.03–0.53)], suggesting a protective effect, but TCRE neither reduced nor increased the risk of EA [SIR = 1.27 (0.86–1.88)] [43] (Table 1). This wide variation in the incidence is likely related to the small number of patients (denominator < 500) in six studies, and the wide variation in follow-up (1.9 to 20 years) and age of women in all studies. Therefore, the summary incidence (0.11%) has to be interpreted with caution.

It should be noted, however, that comparing women having EA with women having LNG-IUS to treat AUB, Dood et al. reported that there was a notable but not statistically significant difference favouring LNG-IUS treatment versus EA based on EC rates of 3/4776 (0.06%) and 1/3558 (0.03%) women in the EA and LNG-IUS groups, respectively [39]. However, in a secondary analysis LNG-IUS had a lower rate of EC than all the other treatments (LNG-IUS HR, 0.12; CI, 0.02–0.83; *p* = 0.03) [39]. Dood’s study supports the hypothesis that EA might reduce the risk of EC since it is equivalent to the risk reduction provided by LNG-IUS which has been shown to be the most effective prevention measure for EC. Large-scale observational studies describe up to a 78% reduction in EC risk among LNG-IUS users if used long-term [47,48], especially in women with a BMI ≥ 40 [49,50]. Applying the same analogy, the apparent reduction in incidence of EC after EA allows speculation that EA might be considered an appropriate preemptive minimally invasive approach to potentially reduce the risk of EC in high-risk women with or without AUB.

In the last 30 years, the incidence of EC worldwide has increased by 132% and is set to continue to rise in response to an ageing population, and increasing global rates of obesity and diabetes, all being risk factors for EC [51]. The increased prevalence of obesity in women and its association with increased risk of EC [7,8], together with the widespread utilization of EA to treat AUB in these women, raises the possibility of a higher incidence of EC in obese women undergoing EA than in the general population.

Moreover, based on the short follow-up and the median age of 54 at PAEC diagnosis (10 years earlier than the median age diagnosed with EC in the general population) in our review, the incidence of PAEC likely will increase as these women age and reach the end of their lives. Women aged 50–70 years have a two-to three-fold greater risk relative to women < 50.

Regardless of the significant heterogeneity of the studies and the short and widely ranged follow-up, we hypothesize that, in the short term, EA may have a protective effect since the life-time risk of EC in the general population is 2 to 3% compared to approximately 1/1000 following EA. The risk reduction is likely due to a dual effect of EA reducing endometrial volume and also destroying/eliminating occult pre- or malignant endometrial foci. The latter mechanism is supported by the fact that no residual cancer was found in hysterectomy specimens in several cases in the present review (Table 2, 9 of 12; Table 4, two of 16 cases) and in prior publications [3,4,5]. Based on similar experience, we have reported that hysteroscopic EA can be an effective definitive treatment in selected women with AUB and AEH and even EC in women who refuse or are at high risk for hysterectomy and are compliant with appropriate surveillance and regular long-term follow-up [3].

In our review, the method of EA was not always clearly articulated to determine if one particular method is more effective in altering EC risk. Flöter Rådestad et al. reported that there was a significant reduction in EC after REA, suggesting a protective effect, whereas after TCRE the incidence was within the expected rate in the general population [43]. This discrepancy may be due to patient selection and characteristics and the dependence of TCRE on the skill and experience of the surgeon, all of which can result in resecting variable endo-myometrial depth. Thus, TCRE does not always remove a deeper endometrial pocket such as adenomyosis, leaving more residual endometrium than all other methods applying heat energy and destroying more and deeper myometrium. Dood et al. did not find any difference in EC incidence when comparing first- and second-generation EA methods, with medical management [39]. The discrepancy in outcomes after different methods of EA likely reflects variations in patient characteristics, patient selection, surgical technique, EA method used and surgeon’s experience and/or expertise.

Our review also underscores the importance of intra-operative endometrial sampling, especially during nonresectoscopic EA methods, since at least 12 cases of EC were identified by additional sampling during EA and treated appropriately, although all had benign pre-EA endometrial biopsy (Table 2). Interestingly, although hysterectomy was delayed 34 and 60 months in two patients, the EC was stage IA, G1-2 in both cases.

To minimize the likelihood of missing significant intrauterine pathology in women undergoing EA, we advocate and perform total TCRE (including fundus and tubal ostia) in all women with PMB, in the absence of benign (within 6 months) endometrial biopsy, in the presence of risk factors (including BMI > 30) for endometrial neoplasia, and in the presence of any intrauterine lesions (polyps or fibroids). For patients in whom REA or non-hysteroscopic EA is performed, we advocate and perform hysteroscopy and endometrial curettage immediately prior to EA to evaluate the endometrial cavity, obtain additional endometrial samples, and thin the endometrium. As a result, we have identified several cases of incidental miscellaneous uterine malignancies including undiagnosed EC among 5750 women undergoing primary resectoscopic EA with benign pre-ablation endometrial biopsy performed by the senior author (G.A.V) from 1990 through December 2017 [52].

The value of additional sampling during EA is also underscored in Table 2 (EC likely present during EA), indicating that even in the presence of a pre-EA biopsy showing endometrial hyperplasia, no intra-EA biopsy was done in two women. In the third case the pre-EA biopsy was upgraded from complex to AEH after EC diagnosis, which is a pre- or coexisting lesion with EC in a significant number of cases.

The median age of diagnosis of EC in the general population is 64 years. In the 16 pre-menopausal women who had EA, the median age and (range) of diagnosis of PAEC was approximately 54 (42–66) years. This is calculated from the median age of 47.5 years at EA and the median time to presentation of 6.5 years. Flöter Rådestad et al. reported median times to EC of 3.0 and 8.3 years after TCRE and EA, respectively [43]. Dood et al. concluded that there was no delay in diagnosis of EC when comparing EA versus medical management of AUB [39].

Our review has considerable limitations including a small number of heterogeneous reports with a small number of patients in at least half the studies and short and widely varied follow-up, all of which indicate that the findings should be interpreted cautiously.

On the other hand, our review identified additional cases of PAEC and one more registry and provides more robust evidence supporting the findings of a recent review by Oderkerk et al. reporting on 11 studies involving 38 EC among 29,102 patients with a history of EA. The authors found that the incidence of endometrial cancer ranged from 0.0% to 1.6%—similar to our range. Among these cases, vaginal bleeding was the presenting symptom in 71% of patients, endometrial sampling was successful in 89% of the described cases and 90% of the pathology showed early-stage endometrioid adeno-carcinoma (FIGO Stage I). The authors concluded that previous EA is not associated with the development of EC, diagnostic work-up is not impeded by previous EA and EC after endometrial ablation are not detected at an advanced stage [46].

## 5. Summary Statements and Recommendations

1. The findings of this narrative review should be interpreted with caution since the studies and the data are heterogeneous and limited, and the median follow-up is short with a wide range.
2. The incidence of PAEC appears to be reduced in the short term compared with the incidence in the general population, but the incidence will likely increase as women age and reach the end of their lives.
3. No one method of EA emerged as distinctive in altering the risk of PAEC. In a national registry, REA significantly reduced the risk of PAEC suggesting a protective effect, while the risk after TCRE remained similar to that expected in the general population.
4. The mechanistic effect of risk reduction may be due to a combination of reduced endometrial volume and elimination of occult pre- or malignant endometrial pathology; the latter mechanism is supported by identifying no residual EC in hysterectomy specimens in several cases and other cases reported in the literature.
5. Pre-EA benign endometrial pathology cannot always be relied upon, since endometrial pre- or malignant pathology (AEH and EC) has been identified during EA with benign pre-EA endometrial pathology. Endometrial sampling during EA is recommended in spite of benign pre-EA reporting.
6. EA in women with PMB is appropriate for both diagnosis and treatment in those women with inadequate or difficult-to-perform endometrial biopsy, in the presence of intrauterine lesions (polyps or fibroids), and in women on HRT with AUB unresponsive to hormonal manipulation.
7. The age of presentation of PAEC does not appear to be delayed. Interestingly, in pre-menopausal women, it was approximately 10 years earlier than the average age in the general population.
8. The presentation of PAEC was vaginal bleeding with or without pain in both post- (89%) and pre-menopausal (94%) women which is similar to the presentation of EC in post-menopausal (94%) women in the general population [53].
9. Diagnosis of PAEC was achieved by hysteroscopy and D&C/biopsy in 86% and 93% attempted procedures in the post- and pre-menopausal women, respectively, similar to that reported in the general population [54,55].
10. PAEC was stage I in 78% and 81% in the post- and pre-menopausal women, respectively which is similar to that in the general population-stage I (65–75%); II (5–10%); III (10–15%)*,* and IV (5–10%) [56].

## 6. Conclusions

Based on the available limited evidence, we conclude that the incidence of PAEC is quite variable and EA might decrease the risk of EC in the short term. The mechanistic effect is likely due to a quantitative reduction in endometrial volume and eliminating occult pre- or malignant endometrium which is vulnerable to EA. However, the incidence of PAEC will likely increase as women with EA reach the end of their lives. The mode and time to presentation, diagnostic work-up, including endometrial biopsy and hysteroscopy, and stage of PAEC are not altered by EA. Endometrial sampling during EA identifies additional cases with pre- or malignant endometrium and we advocate that all patients undergoing any method of EA have intra-operative hysteroscopy and endometrial sampling, regardless of benign pre-EA endometrial pathology.

## 7. Future Directions

We propose that EA be evaluated further for long-term effects on PAEC with longitudinal prospective studies. Also, EA should be evaluated specifically and considered as a prevention measure of EC by itself and in conjunction with LNG-IUS in women at high risk for EC with and without AUB.

## Figures and Tables

**Table 1:** Incidence of endometrial cancer (EC) after endometrial ablation (EA). TCRE—transcervical endometrium resection; REA—rollerball endometrial ablation; N/A—nonapplicable; NSS—no statistical significance; HR—hazard ratio; RR—relative risk; SIR—standardized incidence ratio.

Author [Reference]	Study	No. of EA	Follow-Up (Years)	Number of EC	Incidence (%)	Statistics
Panoskaltsis [32]	Prospective	193	Median 6 years (5–8)	0	0	N/A
Neuwirth [33]	Retrospective	466	5063 women-years	2	0.43	NSS
Cooper [34]	RCT	263	6 years (5–7)	1	0.38	N/A
Krogh [36]	Retrospective	367	4037 women-years	3	0.82	NSS
Grochmal [37]	Prospective	287	20 years	0	0	N/A
Cooper [38]	Retrospective	11,299	Median 6.2 years (2.7–10.8)	2	0.02	N/A
Dood [39]	Retrospective	4776	Median 4.1 years (1.9–7.2)	3	0.06	HR = 0.45 (0.15–1.40) *p* = 0.17
Morelli [40]	Retrospective	63	---	1	1.59	N/A
Singh [4]	Retrospective	1521	Median 10 years 19,733 women-years	0	0.00 *	RR = 0.01 (0.0–0.28) *p* = 0.001
Soini [41]	Retrospective	5484	39,892 women-years	3	0.05	IR = 0.56 (0.12–1.64)
Kalampokas [42]	Retrospective	901	21.5 years (18–25)	2	0.22 *	RR = 0.32 *p* = 0.001
Flöter Rådestad [43]	Registry	8670 REA 8623 TCRE	Median 7.1 years (3.1–13.3)	2 25	0.02 * 0.3	SIR = 0.53 (0.03–0.53) for REA * SIR = 1.27 (0.86–1.88) for TCRE
Total	12 Studies	39,795	Median 8.5 years (1.9–25)	43	0.11 (0–1.59)	* Statistical significance

Supplemental Material File S1 provides additional context from the studies listed in Table 1.

**Table 2:** Data of 18 cases of endometrial cancer (EC) present or likely present during endometrial ablation (EA). BMI—body mass index; CH—complex hyperplasia; AEH—atypical endometrial hyperplasia; HRT—hormone replacement therapy; HBSO—hysterectomy and bilateral salpingo-oophorectomy; TCRE—transcervical endometrial resection; REA—rollerball endometrial ablation; RF—radiofrequency; TBEA—thermal balloon endometrial ablation.

Case No. Author [Reference]	Age at EA (yr)	BMI	Pre-EA Biopsy	Type of and Time to EA from Biopsy	Time to and Presentation from EA to Diagnosis and Treatment (HBSO)	Stage of EC
1. Dwyer [9]	38	>30	Secretory	3 months: TCRE-EC	<3 months	No residual EC. I
2. Klein [17]	52	--	Proliferative. Given HRT	3 months: REA. D&C-EC	<1 month	Ovarian mets. II, G3
3. Steed [19]	41	26	Proliferative	2 months: REA/TCRE-EC	3 months	No residual EC. I, G1
4–6. Smith [28]	<50 3 cases	--	Benign	Bipolar RF/D&C-EC	Unknown	No residual EC X 3. I X 3
7–12. [30]	40 48 50 Argall 51 47 53	-- -- -- -- -- --	Benign Benign Benign Benign Benign Unknown	3 months: D&C-EC, G1 1 month: D&C-EC, G1 11 months: D&C-EC, G1 17 months: D&C-EC, G1 1 month: D&C-EC, G1 Unknown: D&C-EC, G2	3 months 2 months 2 months 1 month 34 months 60 months	No residual EC. IA, G1 No residual EC. IA, G1 No residual EC. IA, G1 No residual EC. IA, G1 IA, G1 IA, G2
13. Ramy [12]	38	>40	CH	4 months: REA, no biopsy.	6 months: Bleeding. Hysteroscopy, D&C-EC	IA, G1
14. Baggish [15]	50 PMB	>30	CH	6 months: REA, no biopsy.	6 months: Bleeding. Pap smear—EC cells	I, G1
15. Lee [20]	36	36	CH	1 month: TBEA, biopsy—AEH	2 months: Bleeding, hysterectomy—EC	IA, G1
16. du Toit [11]	39	42	Unknown	TCRE-AEH. No treatment	13 months: Bleeding. Pap smear—AEH cells. HBSO-EC	IA, G1
17. Horowitz [14]	63	>40	CH	REA/D&C-AEH. Refused hysterectomy	14 months: Bleeding, D&C, biopsy of umbilical mass—EC	IV. Died 4 months later
18. Igbal [16]	50	---	CH/AEH Danazol 800 mg	1 month: TCRE-benign endometrium	3 years: Bleeding Hysteroscopy/biopsy—EC.	IB, G2

**Table 3:** Data of 9 women with PAEC having been treated with endometrial ablation (EA) for postmenopausal bleeding (PMB). BMI—body mass index; NAEH—nonatypical endometrial hyperplasia; TCRE—transcervical resection of the endometrium; REA—rollerball endometrial ablation; HBSO—hysterectomy and bilateral salpingo-oophorectomy; HRT—hormone replacement therapy.

Author [Reference]	Age/BMI. Comorbidity	Pre-EA Biopsy	Type EA, Histology	Time to and Presentation After EA	Evaluation and HBSO	Stage
Margolis [13]	55/>30. Diabetes, hypertension	NAEH	REA Benign. Premarin	3 years. Amenorrhea. Urinary stress incontinence	None. HBSO/bladder suspension-EC	IC, G1 >50% invasion
Sagiv [21]	57/? No risks	Proliferative	TCRE Secretory	3 years. Bleeding/pain	Hysteroscopy, biopsy unsuccessful. HBSO-EC	IC, G1 >50% invasion
Gaia [35] Hypertension in all 4 women	63/21 HRT 8 years 60/33 53/39 HRT 3 years 56/34	Benign HRT 10 years Benign Benign NAEH	TCRE Benign D&C only Benign TCRE Benign TCRE NAEH	10 years. Bleeding/HRT 8 years. Bleeding 5 years. Bleeding/HRT 6 years. Bleeding/HRT	Hysteroscopy, D&C-EC Hysteroscopy, D&C-EC Hysteroscopy, D&C-EC Hysteroscopy, D&C-EC	IB, G2–3 IC, G1 IA, G1 IB, G1
Tsafrir [29] Hypertension in both women	66/? 62/?	Benign Benign	TCRE TCRE	6 years, Bleeding 6 years. Bleeding	Biopsy—EC Biopsy—EC	IA III. Died 9 months later
Wortman [31]	56/? Breast cancer	Inactive	TCRE Benign	5 years, Intense pain, no bleeding	No attempt	III. Ovarian metastases

? means (indicating unknown).

**Table 4:** Patient demographics, mode of endometrial ablation (EA), presentation, diagnosis, treatment and stage of PAEC of 16 pre-menopausal women with appropriate indications for EA. BMI—body mass index; HBSO—hysterectomy and bilateral salpingo-oophorectomy; NAEH—nonatypical endometrial hyperplasia; RF—radiofrequency; TBEA—thermal balloon endometrial ablation; PMB—post-menopausal bleeding; GnRH—gonadotropin-releasing agonist.

Author [Reference]	Age/BMI	Comorbidity	Pre-EA Biopsy	Type of EA Biopsy	Time to Presentation	Evaluation and HBSO	Stage
Copperman [10]	51/>30	Diabetes hypertension Colon cancer	NAEH Treated with progestin	Coagulation	5 years. PMB	Biopsy—EC	II/III, G2
Brooks-Carter [18]	50/70	Diabetes hypertension	Proliferative	Coagulation	14 months. Bleeding	Hysteroscopy/biopsy—EC. Refused hysterectomy	--
Areia [22]	48/<30	No risks	NAEH Progestin-10 mg GnRH-a-4 mo	Coagulation Benign	30 months. Pain, no bleeding	No attempt	IB, G1
Le Marrec [24]	48/--	Breast cancer	Proliferative	TBEA Proliferative	6 years. PMB	D&C-EC	IA, G2
AlHilli [25]	47/36	Hypertension	Secretory	Bipolar RF	5 years. PMB	Hysteroscopy/biopsy—EC	IA, G1
Wortman [26]	40/24	No risks	Secretory	Bipolar RF	2 years. Bleeding, pain	Hematometra. TCRE-EC	No residual EC IA, G1
Wortman [31]	41/-- 33/-- 45/24 42/-- 49/--	-- -- -- Diabetes, Sleep apnea --	Proliferative -- -- Proliferative 1y before EA Secretory	Bipolar RF Proliferative Coagulation TBEA Proliferative TBEA Bipolar RF	7 years. Bleeding, pain 17 years. PMB, pain 10 years. PMB 8 years. Bleeding, pain 7 years. PMB, pain	Biopsy failed TCRE-EC Biopsy—EC TCRE-EC Hysteroscopy/Biopsy—EC	IA, G1 No residual EC , G3 I G1 IA, G1 IA, G1
Kalampokas [42]	48/37 39/28	-- --	Benign benign	EA/TCRE EA/TCRE	18 years. PMB 18 years. PMB	Biopsy—EC Biopsy—EC	IA IA
Lopez [23] (Abstract)	50/>30	Hypertension	Proliferative GnRH-a	TCRE Proliferative	2 years, PMB	Hysteroscopy/biopsy—EC	IA, G1
MacMahon [27] (Abstract)	48/34	--	--	TBEA	8 years, PMB	TCRE-EC	IA
Tsafrir [29] (Abstract)	37/<25	No risks	Benign	TCRE	6 years, Bleeding	Biopsy—EC	IB

## Data Availability

Data sharing is not applicable to this article as no new data were created or analyzed in this study.

## Supplementary Materials

The following supporting information can be downloaded at: https://www.mdpi.com/article/10.3390/cancers18081290/s1, Supplementary File S1 Additional context from the studies listed in Table 1.
