# Supplementary material for: Endometrial Cancer Related to Endometrial Ablation: A Narrative Review
Source: Cancers (Basel). 2026 Apr 19;18(8):1290. doi: 10.3390/cancers18081290 (PMC13115090; doi:10.3390/cancers18081290)
Supplement: Supplementary file 1 [file cancers-18-01290-s001.zip › cancers-4202912-supplementary.pdf]

SUPPLEMENTAL INFORMATION FOR ENDOMETRIAL CANCER ASSOCIATED WITH ENDOMETRIAL ABLATION

For additional context, we provide a summary of each study.

Panoskaltsis et al reported on their first 193 women, average age 41 (range 24–56) after TCRE and rollerball endometrial ablation (REA) in UK. At a median follow up of 6 years (range 5-8), there was one case of atypical endometrial hyperplasia (AEH) without evidence of EC [32].

Neuwirth et al examined incidence of EC in 509 women with normal endometrial histology who had hysteroscopic EA in two US centers. Among 466 cases, there were two (0.43%) PAEC in a total of 5,063 woman-years; expected incidence was 1.66 cases in an age-matched group with known length of follow-up from the U.S. SEER data; similar to the expected EC incidence in the general population [33].

Cooper et al reported long-term outcomes following microwave endometrial ablation (MEA) or TCRE in Scotland. Of 263 treated women, 236 returned questionnaires. After a minimum of five and maximum of seven years, 20 women in the MEA (16%) and 33 in the TCRE arm (25%) had hysterectomy including one case (1/263; 0.38%) of EC post-MEA [34].

Krogh et al reported on a survey of 367 Danish women following EA during 1990-1996; 52 had EA twice and one three times using rollerball for the fundal area and TCRE for the remaining cavity. Of these, 141 (47%) had hormonal treatment (HT), 35% of which had HT before EA. At 11 years, 82% had returned questionnaires and three women (0.82%) reported EC in 4,037 women-years. According to the Danish National Health Board, the age-standardized incidence of EC rate was 17/100,000 women at age 45 in 1993, which corresponds to the mean age of the women in the middle of the EA period, providing a calculated expected number of 6.8 EC. They concluded that EA does not increase incidence of EC and HT does not influence the course of events [36].

Cooper et al reported risk of further gynaecological surgery and gynaecological cancer following hysterectomy or EA performed between 1989 and 2006 in a population-based retrospective cohort study in Scotland. A total of 37,120 women had hysterectomy, 11,299 underwent EA without a subsequent hysterectomy and 2,779 underwent EA followed by subsequent hysterectomy. The median (interquartile range) duration of follow-up was 11.6 years (7.9 - 14.8) and 6.2 years (2.7 - 10.8) in the hysterectomy and EA (without hysterectomy) cohorts, respectively. In the EA group, two women (0.02%) developed EC [38].

Dood et al investigated whether EA is associated with increased risk or delayed diagnosis of PAEC compared with medical management in women with AUB in a multi-centered retrospective cohort study in UK, between June 1994 and September 2010. All women underwent TCRE, using second-generation EA devices or medical management. Of 234,721

women, 4,776 underwent EA and 229,945 received medical treatment, including combined estrogen-progestin (30,731), progestin-alone (40,457), levonorgestrel intrauterine system (LNG-IUS, 3,588) or expectant/other nonhormone medications. During a median period of 4.07 years (interquartile range, 1.88 - 7.17), EC developed in 3 (0.06%) women in the EA group and 601 (0.26%) in the medical management group (ablation HR, 0.45; CI 0.15 - 1.40; p = 0.17). This corresponded with a study-specific annual EC incidence rate of 59.6/100,000 women, 19.3/100,000 in women who underwent EA, and 60.3/100,000 in women who received medical management. There was no difference in EC incidence when comparing first-and second-generation EA methods with medical management [33]. Comparing women who underwent EA with women who received the LNG-IUS, there was a notable change in the hazard ratio (ablation HR, 6.04; CI, 0.61–60.2; p = .13). However, this observation was based on EC rates of only 3 of 4 776 and 1 of 3,558 women in the EA and LNG-IUS groups, respectively. In secondary analysis, the LNG-IUS was found to have lower rate of EC than all other treatments (LNG-IUS HR, 0.12; CI, 0.02–0.83; p = .03) [39].

Morelli et al reported one EC in 63 women having annual post-EA follow-up and transvaginal ultrasound between January 2000 and August 2014. All women received TCRE or REA or thermal balloon endometrial ablation (TBEA). Nine (14.3%) patients had PMB. Among those with no bleeding, one exhibited endometrial fluid collection and hysteroscopy plus endometrial biopsy identified EC (IA, G2). No patients with uterine bleeding had EC; bleeding attributed to atrophy [40].

Singh et al reported on a retrospective observational study in UK, including 1,521 women (mean age 48 ± 6.3 years) having EA from January 1994 through December 2011. During 18 years, 1,022 women (67.19 %) had first-, and 499 (32.81 %) second-generation EA; TCRE in 843 (55.42 %), bipolar radiofrequency (RF) in 245 (16.11 %), MEA in 243 (15.98 %). At a median follow-up of 10 years (19,733 women-years), none developed EC compared to 261 women who developed EC during the study period in the Yorkshire Cancer Registry database corresponding to a lifetime risk of EC in the general population of approximately 2-3%. (RR 0.0135; CI 0.0007–0.2801; P = 0.0054) [4].

Soini et al examined risk of EC, breast cancer and hysterectomy rate after EA in a retrospective cohort of 5,484 women with EA at mean age (SD, range) 42 years (4.4, 30-49 in Finland between 1997 and 2014. The primary outcome was cancer incidences in the EA cohort compared with those in the background population of the same age. During the study period, the age standardized incidence rate of EC in Finland was 14.2/100,000 women-years adjusted for age according to the World Standard Population. During follow up of 39,892 women-years, the standardized incidence ratio for EC was 0.56 (CI 0.12–1.64); three (0.05%) observed compared

with five expected cases. Of the three PAEC, two were diagnosed at an early stage and one at unknown stage [41].

Kalampokas et al assessed incidence of EC after EA (TCRE/REA) in a prospective observational cohort study from Aberdeen Royal Infirmary. From February 1990 through December 1997, 901 women had EA at a mean age of 42.3 ± 5.7 years (range 26-50). Of these, 204 (22.6%) had a subsequent hysterectomy for reasons other than EC. The incidence of EC was 0.2% (2/901); calculated as 11.1/100,000 women-years. Each study woman was matched by age to the annual observed incidence of EC in northeast Scotland for each year from the date of EA until 2015. The mean expected incidence for all women and the subgroup with no hysterectomy (695/901, 77.1%) was estimated to be 26.5 and 35.6 occurrences per 100,000 women-years, respectively (P<0.001). The authors concluded that the risk of EC could be significantly reduced but not eliminated by EA [42].

Flöter Rådestad et al reported incidence of EC from the Swedish National Patient and Quality Registry for Gynecological Surgery (SweGCG) including women who had TCRE or REA (mean age 45.1 years) between 1997 and 2017. All women were followed until hysterectomy, diagnosis of EC, or death based on National Cancer and death Registries. The expected number (population-based estimate) of PAEC was 15 cases. During a median follow-up of 7.1 years (interquartile range 3.1 - 13.3 years), the cumulative rates of PAEC were 0.3% (25/8,626) after TCRE and 0.02% (2/8,670) after REA, respectively; giving a standardized incidence ratio of 1.27 (CI 0.86 - 1.88) after TCRE and 0.13 (CI 0.03 - 0.53) after REA. Median times to PAEC were 3.0 and 8.3 years after TCRE and REA, respectively. [43].
